# scFv-Fcs as a Radiotheranostic Platform for Targeting TRA-1-60 in Pancreatic Cancers

**DOI:** 10.21203/rs.3.rs-10592835/v1

**Published:** 2026-08-25

**Authors:** Sajmina Khatun, Farzaneh Rezazadeh, Alexander John Deck, D. Nuwangi Kulasekara, Allen-Dexter Saliganan, Wendy Wiesend, Hyejeong Jang, Steve M. Patrick, Seongho Kim, Nerissa T. Viola

**Affiliations:** Karmanos Cancer Institute, Wayne State University; Karmanos Cancer Institute, Wayne State University; Karmanos Cancer Institute, Wayne State University; Karmanos Cancer Institute, Wayne State University; Karmanos Cancer Institute, Wayne State University; Corewell Health; Karmanos Cancer Institute, Wayne State University; Karmanos Cancer Institute, Wayne State University; Karmanos Cancer Institute, Wayne State University; Karmanos Cancer Institute, Wayne State University

**Keywords:** TRA-1-60, radiopharmaceutical therapy, immunoPET imaging, Zirconium-89, Lutetium-177, tumor stemness, targeted radionuclide therapy

## Abstract

**Purpose:**

TRA-1-60 (TRA) is an established cancer stemness marker implicated in tumor initiation, therapeutic resistance, and disease relapse. It is minimally expressed in normal differentiated tissues, making it an appealing biomarker for immunopositron emission tomography (immunoPET) imaging and radiopharmaceutical therapy (RPT). Full-length monoclonal antibodies (mAbs) often exhibit prolonged circulation, which can lead to increased background signal and potential off-target radiation exposure to normal tissues, thereby raising concerns of blood-associated toxicity. To address these limitations, we developed a smaller TRA-targeted scFv-Fc antibody fragment and evaluated its potential as a unified immuno-theranostic platform for both noninvasive molecular imaging and targeted RPT of TRA^+^ tumor subpopulations.

**Experimental design:**

A TRA-specific scFv-Fc was conjugated with DFO-NCS and radiolabeled with zirconium-89 (t_1_/_2_ ≈ 3.27 days) for immunoPET imaging. *In vitro* uptake of [^89^Zr]Zr-DFO-anti-TRA scFv-Fc demonstrates membrane-bound and internalized accumulation in high TRA-expressing NCCIT embryonal carcinoma cells. *In vivo* imaging was performed in BxPC-3 and AsPC-1 pancreatic xenografts with high and low TRA expression, respectively. Time activity curves were generated to determine the optimum timepoint for imaging. Tissue distribution was investigated in BxPC-3-bearing mice to define the pharmacokinetics of the new imaging agent. For treatment, the scFv-Fc was conjugated to CHX-A″-DTPA and radiolabeled with Lutetium-177 (t_1/2_ ~ 6.67 d). SPECT imaging, biodistribution, and receptor-blocking studies were performed to evaluate the RPT agent’s specificity and accumulation. Therapeutic efficacy in BxPC3 tumor-bearing mice was evaluated using escalating activities (300–750 mCi). Tumor growth was monitored. Toxicity was evaluated through blood chemistry studies and histopathologic analyses of liver, kidney and spleen. TRA expression post-treatment was probed via immunofluorescence while tumor response was further characterized by immunohistochemical (IHC) analysis of Ki-67. Analyses of macrophage recruitment was probed via IHC to establish involvement of innate immunity post-TRA RPT.

**Results:**

[^89^Zr]Zr-DFO-anti-TRA scFv-Fc was produced with high radiochemical purity and stability. Tumor accumulation peaked as early as 24 h p.i. with enhanced contrast achieved between 24–48 h p.i. Minimal uptake was observed in gastrointestinal tissues especially in the pancreas and stomach. Importantly, blood pool residency was significantly decreased at 120 h p.i. ImmunoPET enabled sensitive and specific visualization of TRA^+^ expression with the radiotracer displaying tumor retention up to 120 h p.i. Competitive blocking studies attenuated the uptake of the radiotracer in BxPC-3 tumors, demonstrating specificity. The radiolabeling of the RPT agent [^177^Lu]Lu-DTPA-anti-TRA scFv-Fc exhibited high radiochemical purity. Specific and sustained tumor uptake was confirmed via SPECT imaging and biodistribution. Blocking studies significantly reduced tumor uptake of TRA RPT, confirming specificity. Treatment resulted in dose-dependent tumor growth suppression without significant hematologic or organ toxicity. TRA expression was reduced across all three treated cohorts compared to the untreated group. Histological analyses revealed marked reductions in tumor burden based on Ki-67-defined proliferative activity. Increased infiltration of macrophages was observed in the periphery in treated versus untreated groups, suggesting that the RPT agent elicited an immune response.

**Conclusions:**

We successfully established a TRA-targeted radiotheranostic platform which integrated immunoPET imaging and RPT using an scFv-Fc scaffold. This approach enables non-invasive diagnostic mapping of tumor-specific TRA expression and supports targeted delivery of therapeutic radiation with minimal organ toxicity while effectively suppressing both proliferative tumor subpopulations.

## Introduction

Pancreatic ductal adenocarcinoma (PDAC) remains one of the most lethal malignancies worldwide, characterized by late-stage diagnosis, aggressive biological behavior, and profound resistance to conventional systemic therapies [1, 2]. Despite advances in surgical techniques and chemotherapeutic regimens, the five-year survival rate for PDAC remains, below 12%, underscoring the urgent need for novel therapeutic strategies [3]. A fundamental contributor to this dismal prognosis is intratumoral heterogeneity, the coexistence of phenotypically and functionally distinct tumor cell populations within a single tumor mass [4, 5]. Among these subpopulations, cancer stem cells (CSCs) represent a therapy-resistant compartment, endowed with self-renewal capacity, phenotypic plasticity, and the ability to regenerate tumor bulk following cytotoxic insult [6, 7]. CSCs are strongly implicated in treatment resistance, metastatic dissemination, and disease relapse, yet their selective identification and eradication in the clinical setting remain formidable challenges [2, 8]. Conventional diagnostic modalities, including computed tomography (CT) and magnetic resonance imaging (MRI), provide valuable anatomical information but lack the molecular specificity required to delineate therapy-resistant CSC subpopulations within the tumor microenvironment [9]. Positron emission tomography (PET) enables highly sensitive, noninvasive imaging of biological processes in vivo; however, its effectiveness is largely dependent on the specificity of the radiotracer employed. As the gold standard for cancer staging and diagnosis, [^18^F]FDG is capable of delineating malignancies but primarily reflects glucose metabolism and therefore lacks the specificity needed to identify CSC populations [10]. This highlights a critical unmet need for molecularly targeted imaging agents capable of noninvasively mapping and characterizing CSC-enriched tumor compartments, thereby enabling rational stratification of patients and guiding precision therapeutic interventions.

TRA-1-60 (TRA), a glycosylated sialyl-keratan sulfate epitope associated with the transmembrane protein podocalyxin (PODXL), has emerged as a compelling cell-surface biomarker of CSC-like tumor populations [11, 12]. Originally identified as a pluripotency marker in human embryonic and induced pluripotent stem cells, TRA expression has since been documented across a broad spectrum of human malignancies, including pancreatic, breast, prostate, colorectal, ovarian and testicular cancers [13, 14]. In these contexts, TRA expression correlates with enhanced tumor aggressiveness, increased metastatic potential, chemoresistance, and inferior clinical outcomes [15, 16]. Crucially, TRA expression is largely absent in normal differentiated adult tissues, and its extracellular, cell-surface accessibility renders it particularly amenable to antibody-based targeting strategies [17]. Collectively, these attributes position TRA as a highly attractive molecular target for the development of both diagnostic imaging and therapeutic platforms selectively directed at therapy-resistant tumor subpopulations.

Our group previously developed and evaluated TRA targeted immunoPET imaging agents using the monoclonal antibody Bstrongomab (Bsg) [18]. Radiolabeling of Bsg with zirconium-89 ([^89^Zr], t½ ≈ 3.27d), a radionuclide with a half-life well-matched to the prolonged circulation kinetics of intact antibodies, enabled high-resolution quantitative PET imaging of TRA-expressing tumors in preclinical xenograft models [19, 20]. These studies successfully discriminated high TRA-expressing (BxPC-3) from low TRA expressing (PC-3) tumor models, establishing proof-of-concept for noninvasive, antibody-mediated assessment of CSC-associated tumor heterogeneity [21, 22]. Subsequent expansion of this framework to encompass theranostic applications, integrating [^89^Zr]-based imaging with [^177^Lu]-based therapy, further underscored the translational potential of TRA-directed radiopharmaceuticals for precision oncology [11–13]. Despite these encouraging findings, the clinical deployment of full-length IgG antibodies as imaging and therapeutic agents is constrained by several pharmacokinetic limitations.

To address these limitations, we engineered an antibody fragment with optimized size and pharmacokinetic properties. Building on this work, we co-opted to develop an scFv-Fc (110 kDa). This intermediate-sized format retains the full antigen-binding specificity of the parental antibody while conferring improved tumor penetration, accelerated systemic clearance, and a more favorable pharmacokinetic profile. This intermediate-sized format retains the antigen-binding specificity of the parental antibody while offering improved pharmacokinetic properties [23, 24]. In this study, we developed a TRA-targeted immunotheranostic platform by radiolabeling the scFv-Fc construct for both imaging and therapy. Specifically, the construct was labeled with zirconium-89 for immunoPET imaging and lutetium-177 (t_1/2_ ~ 6.67 d) for radiopharmaceutical therapy, enabling a unified approach for tumor detection and treatment. Using TRA-expressing BxPC-3 pancreatic cancer xenograft models, we evaluated tumor targeting, imaging capability, and therapeutic efficacy. This work establishes a foundation for the development of a TRA-targeted strategy for noninvasive tumor characterization and targeted radiotherapy.

## General Materials & Methods

All reagents and supplies were purchased commercially. All cell lines were purchased from ATCC unless otherwise stated. All data collected during the current study are available from the corresponding author on reasonable request.

### Cell lines and xenografts

The human pancreatic cancer cell lines BXPC-3 and AsPC-1 from ATCC were cultured under standard conditions at 37 °C in a humidified atmosphere containing 5 % CO_2_. The cell lines were maintained in RPMI-1640 growth medium supplemented with 10 % fetal bovine serum and 1 % penicillin/streptomycin. All cultures were regularly screened for mycoplasma contamination using a mycoplasma detection assay (Lonza).

All animal procedures were approved by and performed in accordance with the Wayne State University Institutional Animal Care and Use Committee. All *in vivo* experiments were conducted in strict accordance with Wayne State University IACUC-approved protocols and regulations established by the Division of Laboratory Animal Resources. Tumors were generated by subcutaneous inoculation of 5 × 10^6^ cells on shoulder of 6–8-week-old male athymic nude mice (Envigo RMS), using 150–200 μL of a 1:1 suspension of culture medium and Matrigel while the mice were anesthetized with 1–2% isoflurane in O_2_. Tumor size was measured two to three times weekly using calipers, and tumor volume was determined using the formula for an ellipsoid: Volume = (length × width × height × π)/6. Animals with tumor volumes ranging from 100 to 200 mm^3^ were selected for imaging and therapy studies. General euthanasia procedures were performed via CO_2_ asphyxiation followed by cervical dislocation.

### Antibody conjugation and ^89^Zr radiolabeling

The anti-TRA scFv-Fc was conjugated to p-isothiocyanato-benzyl-desferrioxamine (DFO, Macrocylics) according to published protocols [25]. Briefly, the anti-TRA scFv-Fc in saline, pH~9 was mixed with a solution of 20 mM DFO in DMSO at a 1:5 scFv-Fc:DFO mole ratio. The reaction was incubated for 1 h at 37 °C at 300 rpm. After incubation, excess DFO was removed through centrifugal filtration with a molecular weight cut-off of 30 kDa with saline as the eluent (Zeba). [^89^Zr]Zr-oxalate (1 mCi or 37 MBq, 3D Imaging) was neutralized to a pH range of 7.0–7.2 and reacted with DFO-anti-TRA scFv-Fc (0.2 mg, 2 nmol) and incubated for 1 h at room temperature. The reaction was quenched with 50 mM EDTA. The resulting mixture was then subjected to purification using spin column centrifugation (MWCO ~ 30 kDa). Radio-instant thin layer chromatography (radio-iTLC) was used to analyze the radiochemical purity and yields. Silica gel-impregnated iTLC strip (Agilent Technologies, Cat. No: SGI0001) and 50 mM EDTA were utilized as the solid and mobile phase, respectively. The stability of [^89^Zr]Zr labeled anti-TRA scFv-Fc was assessed by radio-iTLC.

### Lu-177 conjugation and radiolabeling

The anti-TRA scFv-Fc construct was chemically linked to *p*-isothiocyanatobenzyl-cyclohexyl-diethylenetriamine pentaacetic acid (CHX-A″-DTPA; Macrocyclics, Inc.) following established conjugation procedures with minor adaptations [13]. Briefly, CHX-A″-DTPA (33 nmol from a 20 mM stock solution prepared in dimethyl sulfoxide) was introduced into a solution of scFv-Fc prepared in 1× phosphate-buffered saline (PBS, pH ~9). The reaction mixture was incubated at 37 °C for 1 h. Excess, unreacted CHX-A″-DTPA was removed using centrifugal ultrafiltration (7 K MWCO Zeba^™^ Spin Desalting Columns). The resulting CHX-A″-DTPA-anti-TRA scFv-Fc conjugate was subsequently buffer-exchanged in 0.2 M ammonium acetate (pH ~5.5). For radiolabeling, no carrier-added [^177^Lu]LuCl_3_ (462.5 MBq, 12 mCi, Isotopia) was incubated with CHX-A″-DTPA-anti-TRA scFv-Fc (23.8 nmol) conjugate and incubated at room temperature for 1 h. The labeling reaction was purified using similar centrifugal ultrafiltration. Radiochemical yields and purities were assessed using radio-iTLC using 50 mM EDTA as mobile phase.

### *In vitro* binding and internalization assay

Specific binding and internalization of [^89^Zr]Zr-DFO-anti-TRA scFv-Fc were evaluated on high TRA-expressing NCCIT embryonal carcinoma cells (provided by Curemeta, LLC). Approximately 200,000 cells were seeded to a 12-well plate and incubated overnight for adherence. Subsequently, the cells were incubated to the radiolabeled [^89^Zr]Zr-DFO-anti-TRA scFv-Fc (1 μCi, 0.2 ng) in 0.5 mL of medium at 37 °C for 1 h, 2 h, 4 h, and 24 h. To assess nonspecific membrane binding and internalization, cells were co-incubated with >80-fold excess non-radiolabeled anti-TRA scFv-Fc in triplicate for 1 h at room temperature. Following each incubation period, unbound radioactivity was removed, and the cells were rinsed multiple times with 1x phosphate buffered saline (PBS). Subsequently, the cells were washed with 1 mL of 100 mM acetic acid + 100 mM glycine (1:1, pH 3.5) at 4 °C to collect the surface bound activity. The cells were lysed with 1 mL of 1 M NaOH. All washes (media plus PBS, acid and alkaline) were collected in separate tubes and measured for counts using a gamma counter (Perkin Elmer 2480 Wizard 2). The percentages of membrane bound and internalized activity was calculated as the fraction of the total activity from all washes.

### PET imaging with [^89^Zr]Zr-DFO-NCS-scFv-Fc-Bsg

Mice bearing BxPC-3 or AsPC-1 xenografts were intravenously injected with [^89^Zr]Zr-DFO-anti-TRA scFv-Fc (6.66–9.25 MBq, 36–50 ug, 0.36–0.5 nmol) in sterile saline. PET scans (Bruker Albira Si) were obtained between 6–96 h post-injection (p.i.) of [^89^Zr]Zr-DFO-anti-TRA scFv-Fc while the mice were anesthetized via inhalation of 1–2% isoflurane in O_2_. Images were reconstructed through Maximum Likelihood Expectation Maximization (MLEM) in 12 iterations and 0.75 mm voxel resolution and were analyzed by PMOD version 4.3 software. Volumes of interest (VOI) were measured by manually drawing on regions of the tumor and selecting organs given by a variety of planar sections. The mean VOI was calculated and expressed as % injected dose per gram of tumor tissue (%ID/g).

### Biodistribution and blocking studies

Tissue biodistribution and *in vivo* evaluation of both imaging and therapeutic constructs were performed in BxPC3 pancreatic tumor bearing mice. For immunoPET studies, animals received intravenous injections of 0.55 – 0.925 MBq (15–20 mCi) [^89^Zr]Zr-DFO-anti-TRA scFv-Fc in saline. Tumor uptake and organ distribution were assessed at 6–120 h p.i. For RPT agent distribution, separate cohorts received 0.74–0.925 MBq (20–25 mCi) of [^177^Lu]Lu-CHX-A″-DTPA-anti-TRA scFv-Fc and was similarly analyzed for distribution and tumor targeting from 3–120 h p.i. In both studies, specificity of target binding was confirmed using competitive blocking with 100-fold excess non-radiolabeled scFv-Fc administered in separate cohorts. At designated time points, mice were euthanized via methods mentioned above, and blood, tumors, and major organs were harvested, weighed, and analyzed. Tissue-associated radioactivity was measured using a gamma counter (Perkin Elmer Wizard^2^ 2480), decay-corrected, and expressed as % injected dose per gram of tissue (%ID/g).

### SPECT imaging with [^177^Lu]Lu-CHX-A″-DTPA-scFv-Fc and therapy studies

Activities of [^177^Lu]Lu-CHX-A″-DTPA-anti-TRA scFv-Fc ranging from 11.1 – 27.75 MBq (300 – 750 μCi ) (n = 5 per cohort) were administered intravenously in BxPC-3 tumor-bearing mice. A separate group received 200 mg of scFv-Fc (n = 4) while another group was left untreated to serve as control (n = 4). On the other hand, AsPC-1 tumors were treated with 20 – 24 MBq (550 – 650 mCi) of the TRA RPT agent. Animals were monitored for body weight changes. Tumor growth was assessed 2–3´ weekly with volumes calculated based on the method described above. BxPC3 tumor-bearing mice (n = 3) receiving 27.75 MBq of [^177^Lu]Lu-CHX-A″-DTPA-anti-TRA scFv-Fc underwent SPECT/CT imaging at 3, 24, 96, and 120 h post-injection to evaluate *in vivo* tracer distribution and tumor uptake over time. The animals were anesthetized via inhalation of 1–2% isoflurane in O_2_ during imaging acquisitions. Images were reconstructed and analyzed using Inveon Research Workplace software (version 4.1).

### Hematological analysis

Whole blood was collected immediately post-sacrifice into ethylenediaminetetraacetic acid (EDTA)-coated and lithium heparin-coated microcollection tubes (Microvette 300, Cat. No. 3071121). A complete blood count (CBC) analysis was performed using a Vetscan HM5 hematology analyzer (Zoetis Inc., Parsippany, NJ). The instrument was calibrated prior to analysis with a synthetic whole blood reference standard provided by the manufacturer (Zoetis Inc., Parsippany, NJ).

### *Ex vivo* immunohistochemistry and immunofluorescence

Tumors, liver, spleen, and kidneys were harvested immediately after euthanasia based on general methods mentioned above and fixed in 4% formaldehyde. Following fixation, tissues were transferred to 70% ethanol for storage prior to paraffin embedding. Tissue sections (5 μm thick) were prepared and mounted on glass slides, followed by hematoxylin and eosin (H&E) staining for histological evaluation. H&E-stained sections from tumor, liver, spleen, and kidney were examined using a Motic slide scanner to assess potential histological evidence of radiation-induced tissue damage. Paraffin-embedded tissue sections were deparaffinized, rehydrated, and subjected to heat-induced antigen retrieval using citrate buffer (pH 6.0). Endogenous peroxidase activity was blocked, followed by nonspecific blocking and overnight incubation with primary antibodies against Ki-67 (clone: D2H10, Cell Signaling Cat. No. 9027) and CD68 (clone: E3O7V, Cell Signaling Cat. No. 97778). After washing, sections were incubated with appropriate biotinylated secondary antibodies and developed using an avidin-biotin peroxidase system with DAB chromogen. Slides were counterstained with hematoxylin, dehydrated, and mounted for microscopic analysis.

BxPC-3 tumors were sectioned, deparaffinized in xylene, and rehydrated through a graded ethanol series to distilled water. Heat-induced antigen retrieval was performed using citrate-based antigen retrieval buffer (pH 6.0; Dako, Carpinteria, CA) in a pressure cooker according to the manufacturer’s instructions. Following antigen retrieval, tissue sections were allowed to cool to room temperature and then blocked with 2.5% normal horse serum for 1 h at room temperature to minimize nonspecific antibody binding. The sections were subsequently incubated overnight at 4°C with mouse monoclonal anti-TRA-1-60(S) primary antibody (Cell Signaling Technology, Cat. #4746) diluted 1:200 in antibody diluent. After three washes with phosphate-buffered saline (PBS), the sections were incubated with Alexa Fluor^®^ 647-conjugated Goat Anti-Mouse IgG1 secondary antibody (Cell Signaling Technology, Cat. #92228) diluted 1:500 for 1 h at room temperature in the dark. Following additional PBS washes, nuclei were counterstained with DAPI for 5 min. Finally, the sections were mounted using ProLong^™^ Diamond Antifade Mountant (Thermo Fisher Scientific) and fluorescence images were acquired using an Akoya fluorescence slide scanner under identical imaging settings for all samples.

### Statistical analysis

Data were analyzed using Graphpad Prism (version 9.1.0) and R (version 4.4.0) and are presented as mean ± S.D. unless otherwise stated. Data distributions were assessed prior to analysis, and when necessary, data was transformed or analyzed using non-parametric methods. Comparisons between groups were performed using t-test or Man-Whiteny U test, as appropriate, with Holm’s procedure applied to adjust for multiple comparisons when applicable, unless otherwise stated. Longitudinal tumor growth for BxPC-3 and AsPC1 xenografts was analyzed using linear mixed-effects models to account for repeated measurements within animals, and pairwise comparisons of growth slopes were adjusted using Holm’s procedure. Baseline tumor volume was defined as 0 mm^3^ on Day 0 for all animals. Tumor volume measurements for AsPC1 were square-root transformed prior to analysis. Tumor latency was analyzed using Cox proportional hazards model or Firth’s penalized Cox model for sparse data, with Holm’s procedure applied to adjust for multiple comparisons.

## Results

### Radiolabeling and in vitro characterization of [^89^Zr]Zr-DFO-anti-TRA scFv-Fc

Radiolabeling of DFO-anti-TRA scFv-Fc was robust with excellent yields (> 98%) with an achieved specific activity of 180 ± 10 MBq/mg (4.9 ± 0.3 mCi/mg). The *in vitro* stability of the [^89^Zr]Zr-DFO-anti-TRA scFv-Fc was monitored by incubating the tracer at 37°C in saline. The radiotracer remains moderately intact up to 72 h, with > 90% retention as evaluated by iTLC (**Fig. S1**).

**Figure 1.**
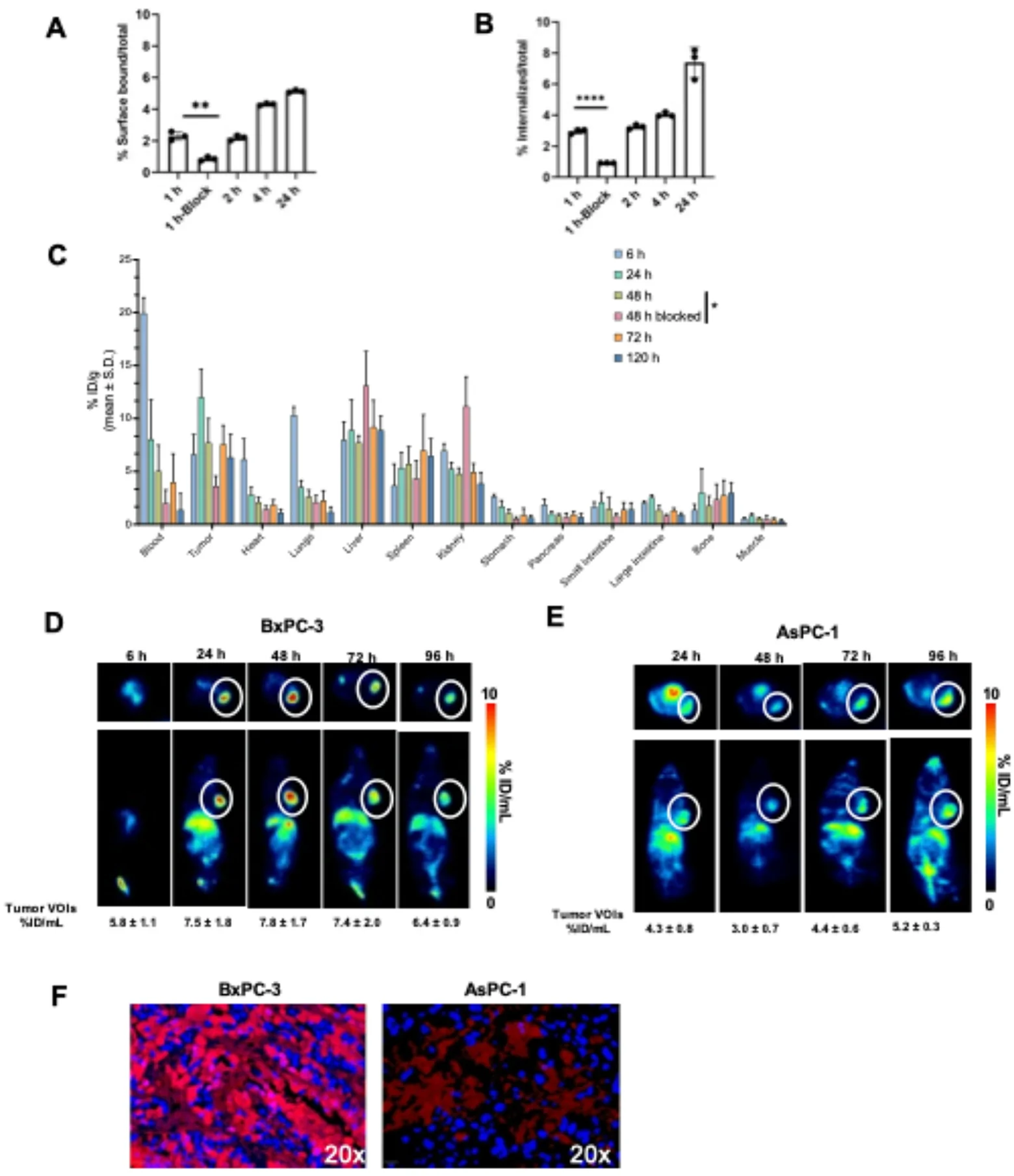
Specificity and distribution of [^89^Zr]Zr-DFO-anti-TRA scFv-Fc. *In vitro* uptake of [^89^Zr]Zr-DFO-anti-TRA scFv-Fc displays **(A)** membranal and **(B)** internalized accumulation in high TRA-expressing NCCIT embryonal carcinoma cells. **(C**) Biodistribution demonstrates accumulation in BxPC-3 tumors with faster blood clearance and minimal uptake in gastrointestinal tissues included the pancreas. Blocking at 48 h displayed lower tracer accumulation in the tumor versus unblocked cohorts establishing specificity. **(D)** *In vivo* PET imaging demonstrates high tracer uptake in BxPC-3 tumors with retention up to 96 h while low accumulation was displayed in the AsPC-1 tumors. **(F)** *Ex vivo* immunofluorescent staining demonstrates elevated TRA expression in BxPC-3 versus AsPC-1 tumors confirming the PET tracer uptake. *P=0.012

Specific binding and internalization of [^89^Zr]Zr-DFO-anti-TRA scFv-Fc with the high TRA-expressing NCCIT cell line was investigated between 1–24 h (Fig. 1A–B). The membrane bound and internalized fractions increased over time. The percentage of membrane binding and internalization of radiotracer at 1 h post incubation was 2.3 ± 0.2 and 2.9 ± 0.1 respectively increasing to 5.1 ± 0.1 and 7.4 ± 0.1% of the totally added activity after 24 h incubation. Co-incubation with excess non-radiolabeled anti-TRA scFv-Fc displayed lower binding and internalization of ^89^Zr]Zr-DFO-anti-TRA scFv-Fc after 1 h incubation (P < 0.001). This data confirms the *in vitro* specificity of the radiolabeled scFv-Fc Bsg to TRA.

### [^89^Zr]Zr-DFO-anti-TRA scFv-Fc exhibits faster blood pool clearance with minimal accumulation in healthy tissue

Tissue distribution of the radiotracer [^89^Zr]Zr-DFO-anti-TRA scFv-Fc was analyzed in BxPC-3-bearing mice from 6–120 h p.i. (**Fig. S2**) Peak tumor uptake was observed at 24 h p.i., reaching 12.0 ± 2.4%ID/g, and was sustained with a gradual reduction to 7.6 ± 1.7%ID/g at 120 h p.i. Notably, blood clearance of [^89^Zr]Zr-DFO-anti-TRA scFv-Fc was significantly faster compared to the ^89^Zr-labeled full-length anti-TRA in both prostate [12] and BxPC-3 tumor models (**Fig. S2**). Minimal uptake was observed in healthy gastrointestinal tissues including stomach, pancreas, and intestines as well as in the bone and muscle across all timepoints. These findings suggest improved pharmacokinetics while retaining tumor specificity of the anti-TRA scFv-Fc radiotracer relative to the full-length antibody. To assess TRA-specific binding, a 100-fold excess of nonradioactive anti-TRA scFv-Fc was administered, leading to a substantial reduction in tumor uptake at 48 h indicating effective inhibition and specificity for TRA.

### [ ^89^ Zr]Zr-DFO-anti-TRA scFv-Fc stratified low versus high TRA-expressing pancreatic cancer xenografts.

To assess tumor uptake and whole-body clearance of [^89^Zr]Zr-DFO-anti-TRA scFv-Fc, PET imaging was performed over time in both BxPC3 (Fig. 1D) and AsPC-1 tumors (Fig. 1E). Tumor uptake was evident as early as 6 h p.i. in BxPC-3 which increased gradually and reached a plateau after 24 h p.i. In contrast, exiguous uptake in AsPC-1 xenografts was observed, likely due to leaky vasculature. This differences in tracer uptake were confirmed via *ex vivo* immunofluorescent staining of TRA where BxPC-3 tumors displayed elevated expression versus AsPC-1 (Fig. 1F, **Fig. S3**). This confirms that the tracer can stratify low versus high TRA-expressing tumors.

### [^177^Lu]Lu-CHX-A”-DTPA-anti-TRA scFv-Fc accumulates in TRA-expressing BxPC-3 pancreatic xenografts with minimal accumulation in healthy tissues

We next radiolabeled the anti-TRA scFv-Fc with lutetium-177, a b^−^ emitting isotope via the bifunctional chelator CHX-A-“-DTPA. Radiolabeling of [^177^Lu]Lu-CHX-A″-DTPA-anti-TRA scFv-Fc was straightforward with high yields and purities (> 97%). The resulting radioconjugate exhibited a specific activity of 166.5 ± 3.7 MBq/mg (4.5 ± 0.1 mCi/mg).

**Figure 2.**
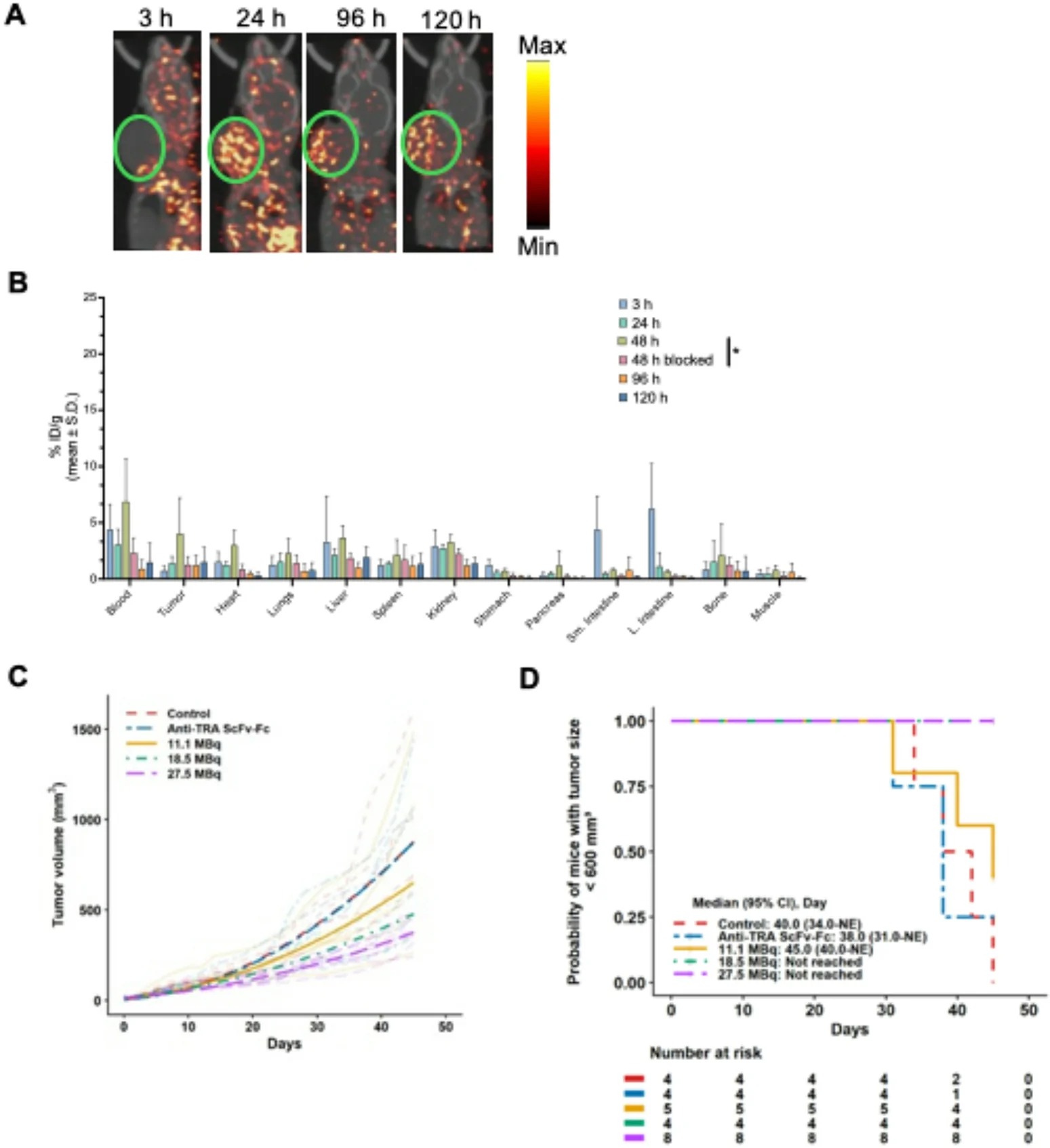
Radiopharmaceutical therapy (RPT) with [^177^Lu]Lu-CHX″-DTPA-anti-TRA scFv-Fc. **(A)** Representative SPECT/CT images of a BxPC-3 tumor-bearing mouse demonstrate prominent tumor accumulation and retention of the RPT agent with progressive clearance from non-target tissues over time. **(B)** Tissue biodistribution of the probe from 3 to 120 h p.i. demonstrated progressive tumor accumulation, with peak uptake observed at 48 h. Co-blocking studies at 48 h confirmed enhanced tumor uptake in the unblocked group compared to the blocked group, indicating target-specific binding. **(C) Tumor volume trajectories for each treatment group over time.** Individual tumor volume trajectories are shown as semi-transparent lines, with solid lines representing model-fitted mean growth curves for each treatment group. **(D) Kaplan-Meier curves for tumor latency across treatment groups.** Tumor latency, defined as the time until tumor volume reached 600 mm^3^. *P=0.016

Representative SPECT images of BxPC-3 tumors exhibited tumor uptake by 24 h with sustained tracer accumulation up until 120 h p.i. (Fig. 2A). Simultaneously, radiotracer activity in non-target organs progressively decreased over time, indicating efficient systemic clearance and favorable *in vivo* pharmacokinetics of the radioconjugate, supporting its suitability for targeted radionuclide therapy of TRA positive tumors.

Tissue distribution studies of [^177^Lu]Lu-CHX-A″-DTPA-anti-TRA scFv-Fc were conducted in BxPC-3 tumor-bearing mice over a time course ranging from 3 to 120 h post-injection (p.i.) to evaluate pharmacokinetics, tumor targeting, and clearance (Fig. 2B). Tumor uptake increased over time, reaching a peak at 48 h p.i. (4.02 ± 1.6%ID/g), followed by a gradual decline while remaining detectable at 120 h p.i. (1.49 ± 0.27%ID/g), indicating moderate tumor retention. Importantly, blood activity declined rapidly, demonstrating faster systemic clearance, consistent with the smaller scFv-Fc format and reduced circulation time. To confirm antigen targeting, a blocking study was performed using a 100-fold excess of non-radiolabeled anti-TRA scFv-Fc, which resulted in a marked reduction in tumor uptake at 48 h p.i. (reduced to ~ 1.3%ID/g, P = 0.0159), confirming specific and saturable binding to the target antigen. Collectively, these results demonstrate enhanced tumor selectivity, improved blood clearance, and favorable pharmacokinetics of [^177^Lu]Lu-CHX-A″-DTPA-anti-TRA scFv-Fc, supporting its potential as an effective targeted radiotherapeutic agent compared with conventional full-length antibody constructs.

### [^177^Lu]Lu-CHX-A″-DTPA-anti-TRA scFv-Fc suppresses tumor growth in high but not low-expressing TRA tumors

The therapeutic effect of [^177^Lu]Lu-CHX-A″-DTPA-anti-TRA scFv-Fc was next evaluated to determine if this antibody fragment can delay tumor growth (Fig. 2C). Linear mixed-effects modeling demonstrated significantly reduced tumor growth rates in all radioligand-treated groups compared with both control and anti-TRA ScFv-Fc groups (all adjusted P ≤ 0.0002; **Table 1**). Growth inhibition increased with activity levels, with the 18.5 MBq and 27.75 MBq groups exhibiting significantly lower growth rates than the 11.1 MBq group, whereas no significant difference was observed between the 18.5 MBq and 27.75 MBq groups (adjusted P = 0.1318). The anti-TRA ScFv-Fc group did not differ from the control group (adjusted P = 0.7256). Kaplan-Meier analysis demonstrated prolonged tumor latency with increasing radioligand dose (Fig. 2D). Median latency was not reached in the 18.5 MBq and 27.75 MBq groups during follow-up. After adjustment for multiple comparisons, only the 27.75 MBq group showed significantly prolonged tumor latency compared with both the control group (adjusted P = 0.0128) and the anti-TRA ScFv-Fc group (adjusted P = 0.0336), corresponding to a substantially lower hazard of tumors reaching 600 mm^3^ (**Table 2**).

Separately, in low TRA-expressing AsPC-1 xenografts, tumor growth trajectories differed significantly between treated and untreated groups with the treated group exhibiting a greater rate of increase in tumor volume over time than the control group (difference in slope – 0.05 ± 0.02 SE; P = 0.0268, linear mixed-effects model) (**Fig. S4A**). However, tumor latency did not differ significantly between groups (HR = 2.10, 95% CI: 0.48–9.11; P = 0.322), despite a shorter estimated median time to tumor volume of > 600 mm^3^ in the treated group (60 vs. 71 d) (**Fig. S4B**).

### Ex vivo validation establishes activity-dependent mitigation of TRA expression and cellular viability

**Figure 3.**
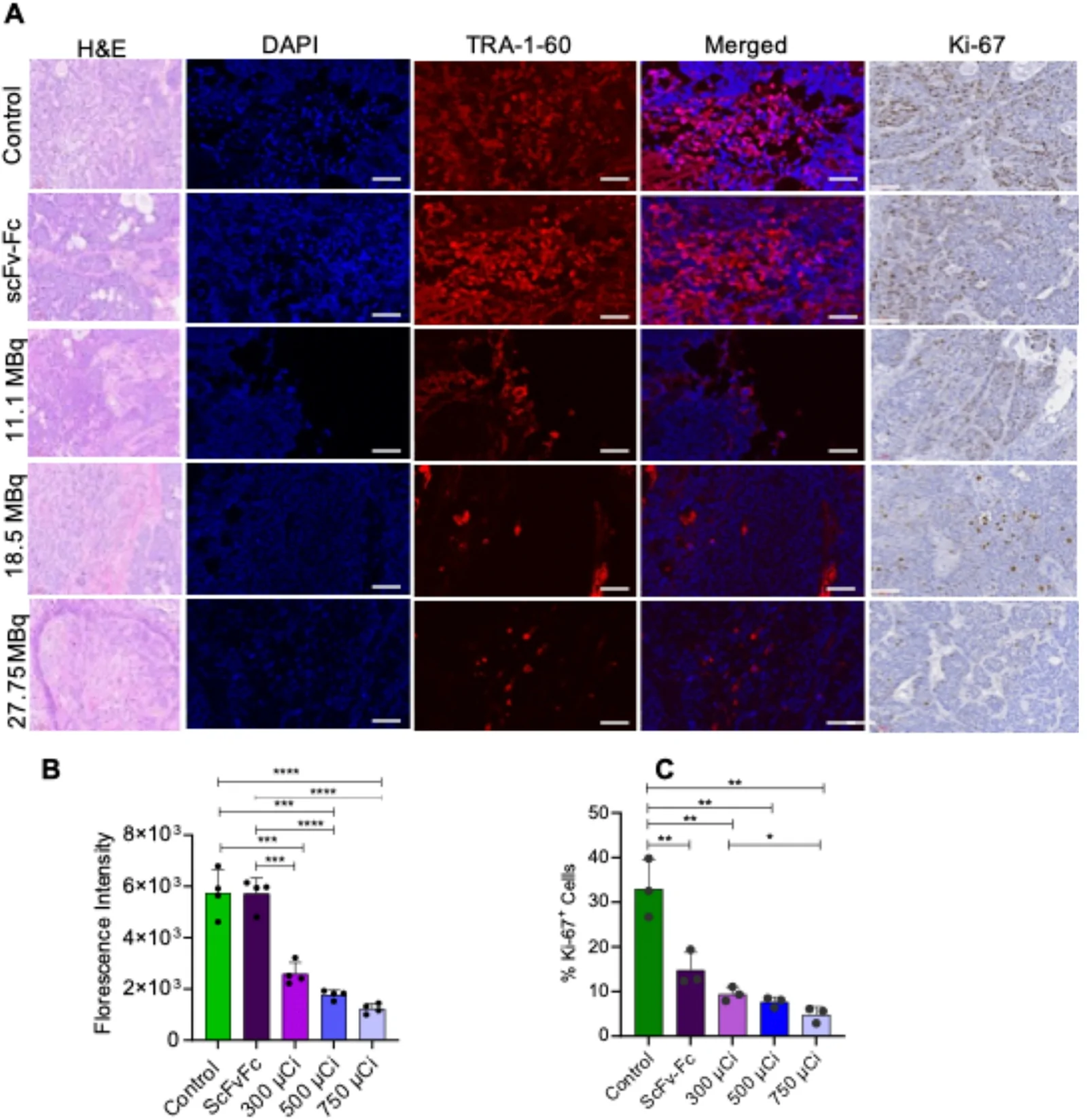
*Ex vivo* validation displays mitigated TRA expression and cell viability in treated BxPC-3 tumors. (A) H&E (20× magnification), nuclear stain using DAPI, immunofluorescence staining of TRA expression and Ki-67 proliferative activity of BxPC-3 tumor sections collected at study termination. Representative Ki-67 immunostaining images demonstrating decreased cellular proliferative activity in a dose-dependent manner. Quantitative analysis of (B) TRA expression and (C) Ki-67 expression demonstrated a dose-dependent decrease in expression and proliferative activity respectively. H&E, Ki67 scale bar: 60 μm. DAPI and TRA IF scale bar: 20 μm. *P = 0.0425, *P = 0.0010, ***P < 0.0001.

Histological examination of tumor sections by H&E staining demonstrates a clear reduction in tumor burden following RPT treatment, as evidenced by disrupted tumor architecture, reduced cellular density, and increased areas of acellularity compared to control tumors, which retain densely packed, viable tumor cells with preserved structural organization (Fig. 3A). Treated tumors show loss of cohesive tumor nests and reduced nuclear crowding, consistent with effective tumor cell killing rather than mere growth arrest. Importantly, no compensatory increase in aggressive histological features is observed, indicating that RPT leads to genuine tumor regression.

Consistent with these morphological findings, IHC analysis showed a marked decrease in Ki-67-positive nuclei in RPT-treated BxPC-3 tumors, indicating significant suppression of tumor cell proliferation (Fig. 3A). Notably, this reduction in Ki-67 staining intensity and positive cell density becomes progressively more pronounced with increasing activities, demonstrating a clear activity-dependent inhibition of tumor proliferation. In parallel, TRA expression is substantially reduced in treated groups, with a corresponding decrease in staining intensity and fewer positive cell clusters compared to controls, where strong and heterogeneous TRA expression is observed (Fig. 3A). Quantifications of TRA expression (Fig. 3B) and % Ki67^+^ cells (Fig. 3C) clearly display effective targeting of tumor-associated antigen-expressing cell populations with concurrent suppression of proliferative activity. Collectively, these data demonstrate an activity-dependent therapeutic effect of the [^177^Lu]Lu-CHX-A″-DTPA-anti-TRA scFv-Fc agent, leading to effective reduction of tumor burden and suppression of both proliferation and stemness-associated signaling programs.

### [^177^Lu]Lu-CHX-A”-DTPA-anti-TRA does not induce hematologic, renal and hepatic toxicity

**Figure 4.**
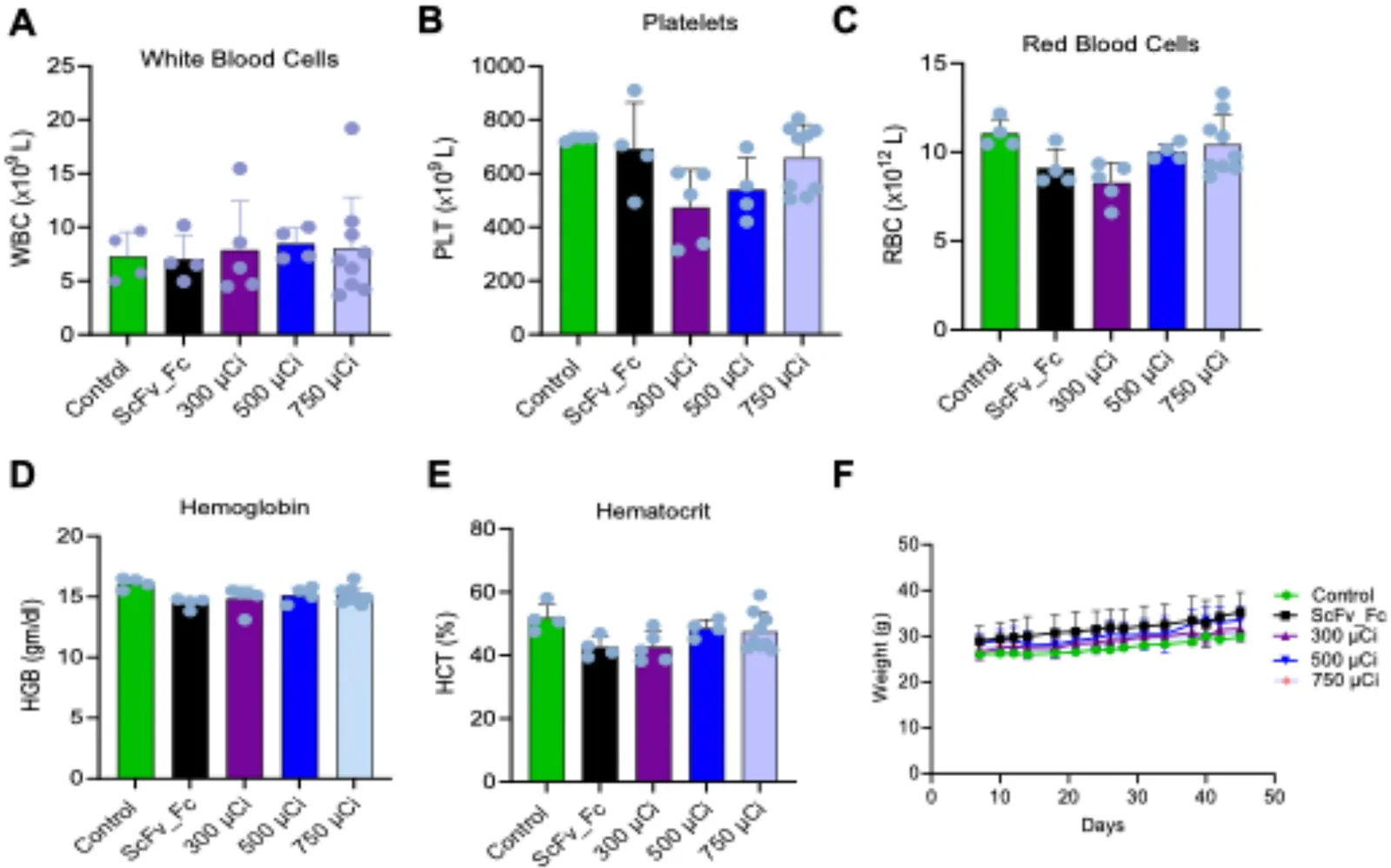
Evaluation of blood toxicity and weight monitoring. Hematological analysis of BxPC-3 xenograft mice treated with [^177^Lu]Lu-CHX″-DTPA-anti-TRA scFv-Fc. Comprehensive blood parameters were evaluated at the study endpoint (maximum tumor volume) for each mouse. **(A)** White blood cells (WBC), **(B)** red blood cells (RBC), **(C)** platelets, **(D)** hemoglobin (HGB), **(E)** hematocrit (HCT), **(F)** Body weight monitoring throughout the study period showed no significant differences between the control and treated groups, suggesting that [^177^Lu]Lu-CHX″-DTPA-anti-TRA scFv-Fc treatment was well tolerated and did not induce observable systemic toxicity.

Hematologic toxicity was evaluated in both control and treated cohorts at the experimental endpoint. Complete blood count analysis revealed no significant reductions in white blood cells (WBC) nor platelet counts in mice treated with all activities of the RPT agent compared to untreated groups (Fig. 4A–B). Consistent with this, no evidence of anemia was observed in treated groups, as demonstrated by comparable red blood cells (RBC), hemoglobin (HGB) and hematocrit (HCT) values (Fig. 4C–E) relative to untreated controls across all evaluated time points. Collectively, these findings indicate that administration of [^177^Lu]Lu-CHX-A″-DTPA-anti-TRA scFv-Fc at therapeutically relevant activities did not induce detectable hematologic toxicity, supporting a favorable safety profile for this RPT approach.

**Figure 5.**
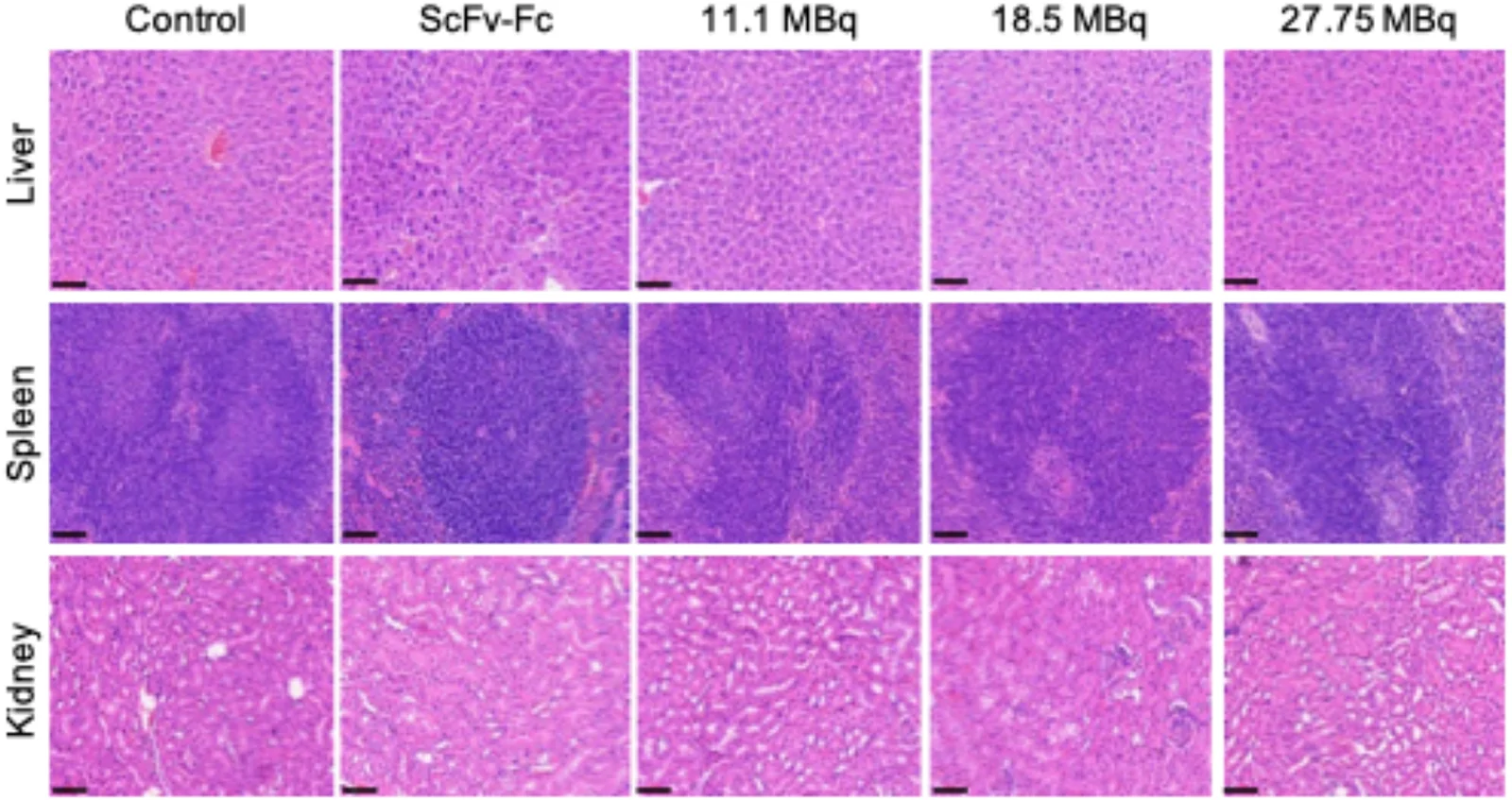
Evaluation of radiation-induced toxicity in the liver, spleen and kidneys. Histological evaluation by H&E staining. Representative sections of liver (top), spleen (middle), and kidney (bottom) from control and treated groups. (H&E, 20× magnification; scale bar: 60 μm).

H&E staining was performed on liver, spleen, and kidney tissues collected from treated and untreated mice to assess for potential radiation-associated tissue injury. Renal sections demonstrated preserved overall architecture, with intact glomerular tufts, normal Bowman’s spaces, and well-organized tubular structures. No evidence of glomerular atrophy or sclerosis, tubular dilation or degeneration, interstitial inflammation, fibrosis, or vascular abnormalities was observed by routine light microscopy, indicating the absence of detectable renal injury within the timeframe examined. Liver sections showed largely preserved hepatic architecture, with well-defined hepatic cords, patent sinusoids, and intact central veins. Hepatocytes appeared morphologically normal, without signs of centrilobular congestion, widespread necrosis, fibrosis, or vascular obstruction. Occasional focal inflammatory changes were noted in isolated samples; however, these findings were limited in extent and lacked morphological features characteristic of radiation-induced liver injury. Spleen tissue exhibited preserved white and red pulp organization, with intact lymphoid follicles and no evidence of architectural disruption, necrosis, or abnormal cellular depletion, suggesting no overt splenic toxicity. Collectively, the findings across all examined organs indicate that treatment with [^177^Lu]Lu-CHX-A″-DTPA-anti-TRA scFv-Fc did not result in observable acute histopathological damage to major dose-limiting organs at the evaluated time points, supporting a favorable tissue safety profile under the conditions studied (Fig. 5)

### TRA RPT modulates the immune microenvironment through macrophage recruitment

**Figure 6.**
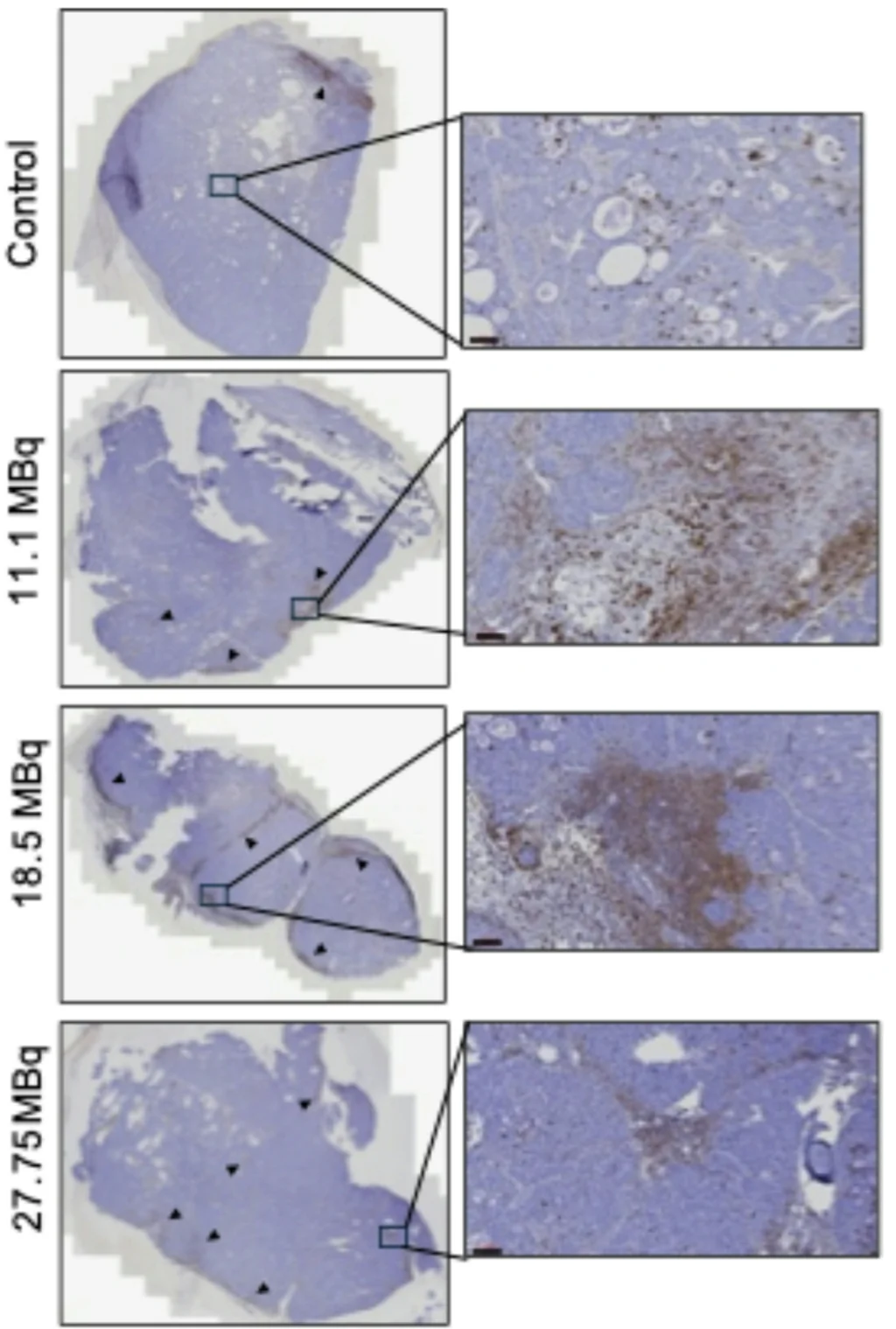
Increased macrophage infiltration is observed in TRA RPT-treated groups. CD68^+^ macrophage (black arrow) infiltration increased in TRA RPT treated cohorts versus control untreated tumors. Infiltration was more pronounced within the tumor periphery. (Magnification: 1× for full tumor section, 10× for inset; Scale bar: 100 μm).

TRA RPT markedly altered the immune microenvironment by enhancing macrophage recruitment in an activity-dependent manner. In the untreated control tumors, only a few scattered CD68^+^ macrophages were detected, indicating minimal basal immune cell infiltration. Following TRA RPT treatment, a substantial increase in CD68^+^ macrophages was observed, with greater accumulation at 11.1 and 18.5 MBq compared with the control group. The 18.5 MBq treatment exhibited the most pronounced macrophage infiltration, characterized by dense CD68^+^ cell populations predominantly localized at the tumor periphery and extending into the tumor parenchyma, suggesting active recruitment of macrophages in response to radiation-induced tissue injury (Fig. 6). Although macrophage infiltration remained elevated in the 27.75 MBq group, the staining appeared less extensive than that observed at the intermediate activity, indicating that the highest activity may differentially influence immune cell recruitment following extensive tumor damage.

## Discussion

In this study, we report the development and preclinical evaluation of a theranostic approach targeting TRA expressing pancreatic cancer cells using an engineered scFv-Fc fragment of Bsg. TRA, a cell-surface marker expressed on pluripotent stem cells and certain cancer stem-like cells (CSCs), represents an attractive target for both imaging and therapy due to its role in tumor initiation, progression, and resistance to conventional treatments. While prior studies with full-length anti-TRA antibody demonstrated specific tumor uptake in TRA-positive models, the large molecular size limited tissue penetration and prolonged systemic circulation, potentially reducing imaging contrast and increasing off-target radiation exposure during therapy. By employing the scFv-Fc fragment, we aimed to leverage a smaller molecular scaffold that maintains antigen specificity while achieving faster clearance and improved tumor-to-background ratios, making it suitable for both PET imaging and radiopharmaceutical therapy (RPT).

Our initial work focused on immunoPET imaging using Zr-89-labeled scFv-Fc. Non-site-specific conjugation via DFO-NCS yielded radiolabeled constructs with high radiochemical yield, excellent *in vitro* stability, and preserved binding affinity. *In vivo* PET imaging in BxPC-3 tumor-bearing mice demonstrated clear and sustained tracer accumulation within tumors, with rapid clearance from non-target organs, confirming efficient and specific TRA targeting in a heterogeneous tumor environment. These findings support the ability of the scFv-Fc antibody fragment to selectively visualize TRA-expressing cells, including potentially rare stem-like populations, while maintaining low background signal.

Building on the imaging data, we developed [^177^Lu]Lu-CHX-A″-DTPA-anti-TRA scFv-Fc for targeted RPT. The radioconjugate demonstrated high radiochemical yield, purity, and stability, ensuring reliable *in vivo* targeting and effective radionuclide delivery. SPECT imaging and biodistribution analyses confirmed antigen-mediated tumor uptake with favorable tumor-to-background contrast; however, comparative evaluation of Zr-89 imaging and Lu-177 distribution suggests subtle differences in retention kinetics, consistent with the expected influence of radionuclide-chelator chemistry and *in vivo* stability on long-term signal persistence. In BxPC-3 xenograft models, treatment resulted in a dose-dependent inhibition of tumor growth; however, this effect was observed up to approximately day 48 of treatment, beyond which tumor growth curves converged with control groups and differences were no longer statistically significant. This attenuation of therapeutic efficacy likely reflects the relatively rapid systemic clearance of the scFv-Fc scaffold, which limits higher tumor accumulation. Notably, residual TRA expression observed even at the highest administered activity (27.75 MBq) further suggests incomplete tumor eradication, supporting the need for repeated dosing or alternative dosing schedules to achieve durable tumor control when using scFv-Fc-based carriers. Together, these findings highlight both the promise and kinetic limitations of the current scFv-Fc platform for RPT. A key limitation of this study is the use of subcutaneous BxPC-3 xenograft models, which do not fully recapitulate the dense desmoplastic stroma, hypovascularity, and immunosuppressive tumor microenvironment characteristic of pancreatic ductal adenocarcinoma (PDAC), potentially influencing delivery and therapeutic response. In addition, the deployment of xenografts and athymic nude mouse strains precluded the extensive evaluation of anti-tumor immunity in tandem with TRA RPT. Collectively, these data underscore the need for optimized dosing strategies and more physiologically relevant tumor models to better predict clinical translation of TRA-targeted theranostic approaches.

Histological and immunohistochemical analyses revealed reduced Ki-67 expression and decreased TRA positive cell populations in treated tumors. This dual effect suggests that TRA targeted RPT not only inhibits proliferating tumor cells but also selectively eliminates stem-like, therapy-resistant compartments that are implicated in tumor recurrence and metastasis. Even though TRA expressing cells may represent a minor fraction of the total tumor mass, their ablation led to significant suppression of overall tumor proliferation, emphasizing the potential of targeting CSCs to improve therapeutic outcomes. Safety assessments demonstrated that [^177^Lu]Lu-CHX-A″-DTPA-anti-TRA scFv-Fc was well tolerated, with no evidence of hematologic or histopathologic toxicity in major organs, including liver, kidney, and spleen. Blood counts remained within normal ranges, and organ architecture was preserved, indicating a favorable therapeutic index for this approach. These findings suggest that scFv-Fc-based TRA RPT can deliver therapeutic radiation doses selectively to tumors while minimizing systemic side effects-a critical consideration for clinical translation. Although complete tumor eradication was not achieved, our results establish proof-of-principle that TRA targeted RPT can selectively ablate stem-like tumor populations, reduce tumor proliferation, and potentially prevent disease recurrence. Future studies may explore fractionated dosing, combination with standard-of-care chemotherapy, or alpha-emitting radionuclides to further enhance therapeutic efficacy while maintaining safety. Additionally, expanding this approach to other cancer models and examining long-term outcomes will provide deeper insight into TRA role in treatment response and tumor progression.

The increased infiltration of CD68^+^ macrophages following TRA RPT suggests that targeted radionuclide therapy not only induces direct tumor cell killing but also promotes remodeling of the tumor immune microenvironment through radiation-induced inflammatory signaling. Radiation-mediated release of damage-associated molecular patterns (DAMPs), cytokines, and chemokines likely facilitates macrophage recruitment to sites of tumor injury, where these cells contribute to the clearance of dying tumor cells, antigen processing, and orchestration of subsequent immune responses. We observed enhanced CD68^+^ macrophages recruitment following TRA RPT treatment which may represent an important mechanism by which TRA RPT enhances anti-tumor immune activation and may provide a rationale for combining TRA RPT with macrophage-directed immunotherapeutic strategies.

## Conclusion

Our study establishes TRA targeted scFv-Fc as a versatile theranostic platform for pancreatic cancer, enabling both sensitive immunoPET imaging and selective radiopharmaceutical ablation of stem-like tumor cells. By effectively targeting TRA positive populations, [^177^Lu]Lu-CHX-A″-DTPA-anti TRA scFv-Fc suppresses tumor proliferation, reduces the therapy-resistant CSC compartment, and demonstrates a favorable safety profile *in vivo*. These findings highlight the potential of TRA directed RPT to overcome therapeutic resistance, limit disease recurrence, and complement standard-of-care treatments, providing a strong rationale for further preclinical optimization and future clinical translation in TRA expressing tumors. Future studies combining TRA-targeted ablation with standard treatment modalities will be explored to achieve enhanced durable responses in TRA expressing malignancies.

## Supplementary Material

Supplementary Files

This is a list of supplementary files associated with this preprint. Click to download.

• SupplementaryFigures071426.pdf

• Table1and2.docx

## Data Availability

All data collected during the current study are available from the corresponding author on reasonable request.
